# Members of the Fibroblast Growth Factor Receptor Superfamily Are Proteolytically Cleaved by Two Differently Activated Metalloproteases

**DOI:** 10.3390/ijms22063165

**Published:** 2021-03-20

**Authors:** Garima Dixit, Willow Schanz, Benjamin A. Pappas, Thorsten Maretzky

**Affiliations:** 1 Inflammation Program, Roy J. and Lucille A. Carver College of Medicine, University of Iowa, Iowa City, IA 52242, USA; garima-dixit@uiowa.edu (G.D.); schanzwillow@gmail.com (W.S.); benjamin-pappas@uiowa.edu (B.A.P.); 2 Department of Internal Medicine, Roy J. and Lucille A. Carver College of Medicine, University of Iowa, Iowa City, IA 52242, USA; 3 Immunology Graduate Program, Roy J. and Lucille A. Carver College of Medicine, University of Iowa, Iowa City, IA 52242, USA; 4 Holden Comprehensive Cancer Center, University of Iowa, Iowa City, IA 52242, USA

**Keywords:** a disintegrin and metalloprotease 10 (ADAM10), a disintegrin and metalloprotease 17 (ADAM17), fibroblast growth factor receptor (FGFR), epidermal growth factor receptor (EGFR), receptor tyrosine kinase (RTK)

## Abstract

Fibroblast growth factor receptors (FGFRs) are a family of receptor tyrosine kinases that have been associated not only with various cellular processes, such as embryonic development and adult wound healing but also enhanced tumor survival, angiogenesis, and metastatic spread. Proteolytic cleavage of these single-pass transmembrane receptors has been suggested to regulate biological activities of their ligands during growth and development, yet little is known about the proteases responsible for this process. In this study, we monitored the release of membrane-anchored FGFRs 1, 2, 3, and 4 in cell-based assays. We demonstrate here that metalloprotease-dependent metalloprotease family, ADAM10 and ADAM17. Loss- and gain-of-function studies in murine embryonic fibroblasts showed that constitutive shedding as well as phorbol-ester-induced processing of FGFRs 1, 3, and 4 is mediated by ADAM17. In contrast, treatment with the calcium ionophore ionomycin stimulated ADAM10-mediated FGFR2 shedding. Cell migration assays with keratinocytes in the presence or absence of soluble FGFRs suggest that ectodomain shedding can modulate the function of ligand-induced FGFR signaling during cell movement. Our data identify ADAM10 and ADAM17 as differentially regulated FGFR membrane sheddases and may therefore provide new insight into the regulation of FGFR functions.

## 1. Introduction

**Figure 1:**
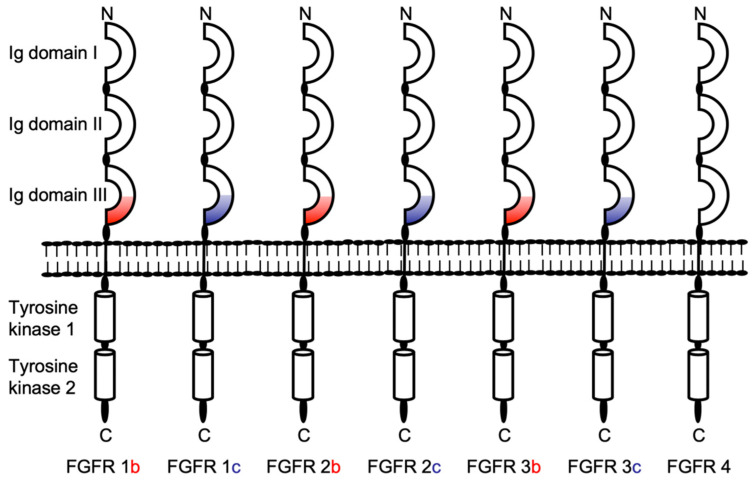
Schematic overview of the structure of the fibroblast growth factor receptor family of receptor tyrosine kinases. FGFRs are highly conserved transmembrane proteins containing an extracellular ligand-binding domain, a transmembrane domain, and a cytoplasmic kinase domain with two adjacent tyrosine residues. The extracellular ligand-binding domain contains three immunoglobulin (IG)-like domains, Ig I, Ig II, and Ig III, which are important for receptor dimerization. Alternative splicing of the second half of the third Ig-like domain gives rise to alternative IIIb (red) or IIIc (blue) isoforms of FGFRs 1 to 3. The proteins coded by FGFRs 1 and 2 can also alternatively produce additional splice variants, generating truncated isoforms [5].

Fibroblast growth factor receptors, a family of high-affinity cell surface receptor tyrosine kinases (RTKs), are essential regulators of critical cellular processes such as cell proliferation and differentiation and have important roles in embryonic development and cancer [1,2,3]. These single-pass transmembrane enzymes have a similar molecular architecture (Figure 1), with a ligand-binding region in the extracellular domain, a single transmembrane helix, and a cytoplasmic region that contains the protein tyrosine kinase domain, which mediates activation through tyrosine phosphorylation [3,4].

Ectodomain shedding of RTKs has been shown to be a posttranslational modification to rapidly and irreversibly regulating cell surface expression levels [6,7,8]. This regulated mechanism liberates the extracellular domain of the receptor through juxtamembrane proteolysis [9], and is distinct from downregulation of the cell surface receptor by internalization or secretion of soluble receptor splice variants lacking a transmembrane domain. Soluble forms of FGFR1 have been identified in the retina and have been suggested to regulate the bioavailability of FGFs and thus their potential as neurotrophic factors [10]. Moreover, FGF-induced cleavage of FGFR1 results in the subsequent release of its C-terminal fragment and translocation to the nucleus, specifically in invasive breast cancer cells [11]. Additionally, soluble FGFRs have also been identified in multiple biological fluids as well as in the extracellular matrix of vascular endothelial cells [12,13,14]. Secretion of these FGFRs can be initiated by the translation of alternatively spliced transcripts and through ectodomain shedding of the transmembrane-bound receptors [15]. However, the nature of the FGFR ectodomain-generating proteases is unknown.

Ectodomain shedding is mainly executed by the a disintegrin and metalloprotease (ADAM) family of metalloproteases. ADAMs are members of the zinc protease superfamily and combine both cell adhesive and catalytic properties. They perform important functions including fertilization, angiogenesis, and wound healing [1,16]. Catalytically active ADAMs including 9, 10, 12, 15, and 17 are widely expressed in somatic tissues and cells (e.g., mouse embryonic fibroblasts (mEFs) and COS-7 cells) [17,18,19,20,21,22,23,24,25]. They are involved in the shedding of various membrane-bound molecules, including cytokines, growth factors, and adhesion molecules [17,19,26]. ADAM10 and ADAM17 have been studied specifically in the context of ectodomain shedding. They are involved in the proteolytic cleavage of various transmembrane proteins such as Notch, EGFR ligands, interleukin 6 receptor, and L selectin [17,27,28,29].

The main goal of the current study was to evaluate what role, if any, ADAMs have in the ectodomain shedding of FGFR family members. We demonstrate that metalloprotease-dependent cleavage of FGFRs 1–4 is mediated by two distinct members of the disintegrin and metalloprotease family, ADAM10 and ADAM17. Loss- and gain-of-function studies in murine embryonic fibroblasts showed that constitutive shedding as well as phorbol-ester-induced processing of FGFRs 1, 3, and 4 is mediated by ADAM17. In contrast, treatment with the calcium ionophore ionomycin stimulated ADAM10-mediated FGFR2 shedding. This shedding process modulates FGF7/FGFR2-mediated cell migration and suggests that soluble forms of FGFRs can act as antagonists, preventing interaction of the membrane-bound forms of FGFRs with their ligands.

## 2. Results

### 2.1. Ectodomains of Membrane-Anchored FGFRs Are Shed by a Metalloprotease Activity

Previous studies have shown that the extracellular domains of all four FGFR family members can be released through membrane-proximal cleavage, leading to generation of C-terminal fragments and soluble N-terminal fragments (NTFs) [15,16,30]. This observation prompted us to evaluate potential involvement of metalloproteases in shedding of these RTKs in cell-based assays. To increase sensitivity of detection of cell surface-bound receptor and shed forms, we used expression plasmids encoding full-length FGFRs 1c, 2c, 3c, and 4 N-terminally fused to an alkaline phosphatase (AP) module, such that the AP module was present in the extracellular domains of the FGFRs and was released into the cell culture medium after shedding [23]. Metalloprotease activity can usually be inhibited by hydroxamate-based compounds [31]. Thus, we tested whether shedding of AP-tagged FGFRs was sensitive to this type of inhibitor.

**Figure 2:**
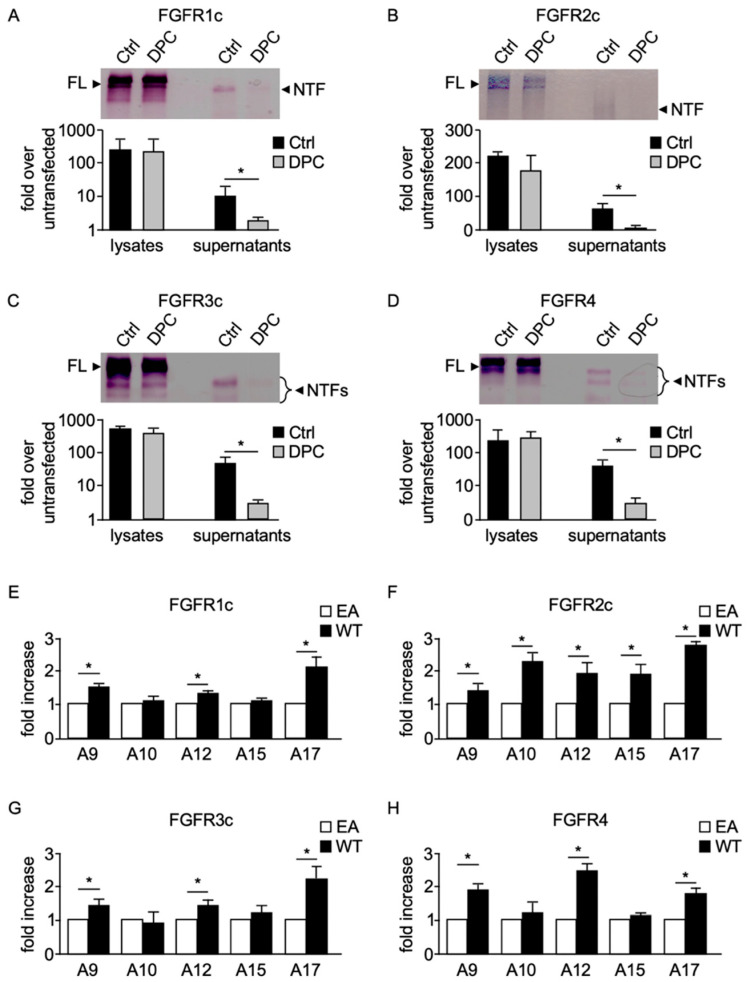
Involvement of a metalloprotease in the constitutive release of FGFRs. In-gel detection of AP-tagged FGFRs 1c (**A**), 2c (**B**), 3c (**C**), and 4 (**D**) in cell lysates (full-length, FL) and supernatants (N-terminal fragments, NTFs) of COS-7 cells. In all cases, there was a significant decrease in release of FGFR-NTFs from cells treated with 5μM of the metalloprotease inhibitor DPC333 (DPC) compared with cells treated with dimethyl sulfoxide (Ctrl). A quantification of three independent experiments is shown in the lower panels of (**A**–**D**). COS-7 cells were co-transfected with wild type (WT) ADAMs 9, 10, 12, 15, or 17 or their catalytically inactive forms harboring an inactivating point mutation in their catalytic site (EA) together with AP-tagged FGFR1c (**E**), 2c (**F**), 3c (**G**), and 4 (**H**). (*) indicates a significant inhibitory effect of DPC (**A**–**D**), or a significant increase in release of FGFR in WT sample compared with EA sample (**E**–**H**). *n* = 3; values are ±SEM; Student’s *t*-test; * *p* ≤ 0.05.

In-gel AP staining of supernatants of AP-FGFR-expressing COS-7 cells demonstrated that the release of FGFR1c (Figure 2A), 2c (Figure 2B), 3c (Figure 2C), and 4-NTFs (Figure 2D) could be strongly reduced by the hydroxamate-based metalloprotease inhibitor DPC333 (DPC), indicating that the majority of released FGFRs in COS-7 cells can be attributed to metalloprotease activity. To confirm transfection efficiency, cell pellets were also analyzed for FGFR expression (Figure 2A–D, top). A densitometric analysis of three independent experiments confirmed that metalloproteases contribute to constitutive shedding of FGFRs (Figure 2A–D, bottom).

This observation, especially when combined with the fact that other RTK substrates are cleaved by similar proteases [8], suggested to us that a member of the disintegrin and metalloprotease (ADAM) family could be involved in the ectodomain cleavage of FGFRs. Since several ADAMs tested to date have activity toward one or more substrates in cell-based assays when compared with their catalytically inactive control [21,25], we tested whether five full-length membrane-anchored catalytically active members of the ADAM protein family had the capacity to shed the ectodomain of FGFRs when overexpressed in cells. We therefore performed “gain-of-function” overexpression studies, in which wild type (WT) ADAMs 9, 10, 12, 15, and 17 or, as a control, their catalytically inactive forms, were co-transfected together with one of the AP-tagged FGFRs in COS-7 cells. Overexpression of ADAMs 9, 12, and 17 increased release of all four FGFRs compared with cells overexpressing inactive (EA) ADAM mutants (Figure 2E–H). In contrast, ADAM10 had no effect on release of AP-tagged FGFRs 1c, 3c, and 4 when overexpressed (Figure 2E,G,H), but significantly increased release of FGFR2c (Figure 2F). Overexpression of ADAM15 only increased release of FGFR2c (Figure 2F), but did not affect release of any of the other FGFR family members.

### 2.2. ADAMs 10 and 17 Are Involved in the Constitutive Shedding of Membrane-Anchored FGFRs

**Figure 3:**
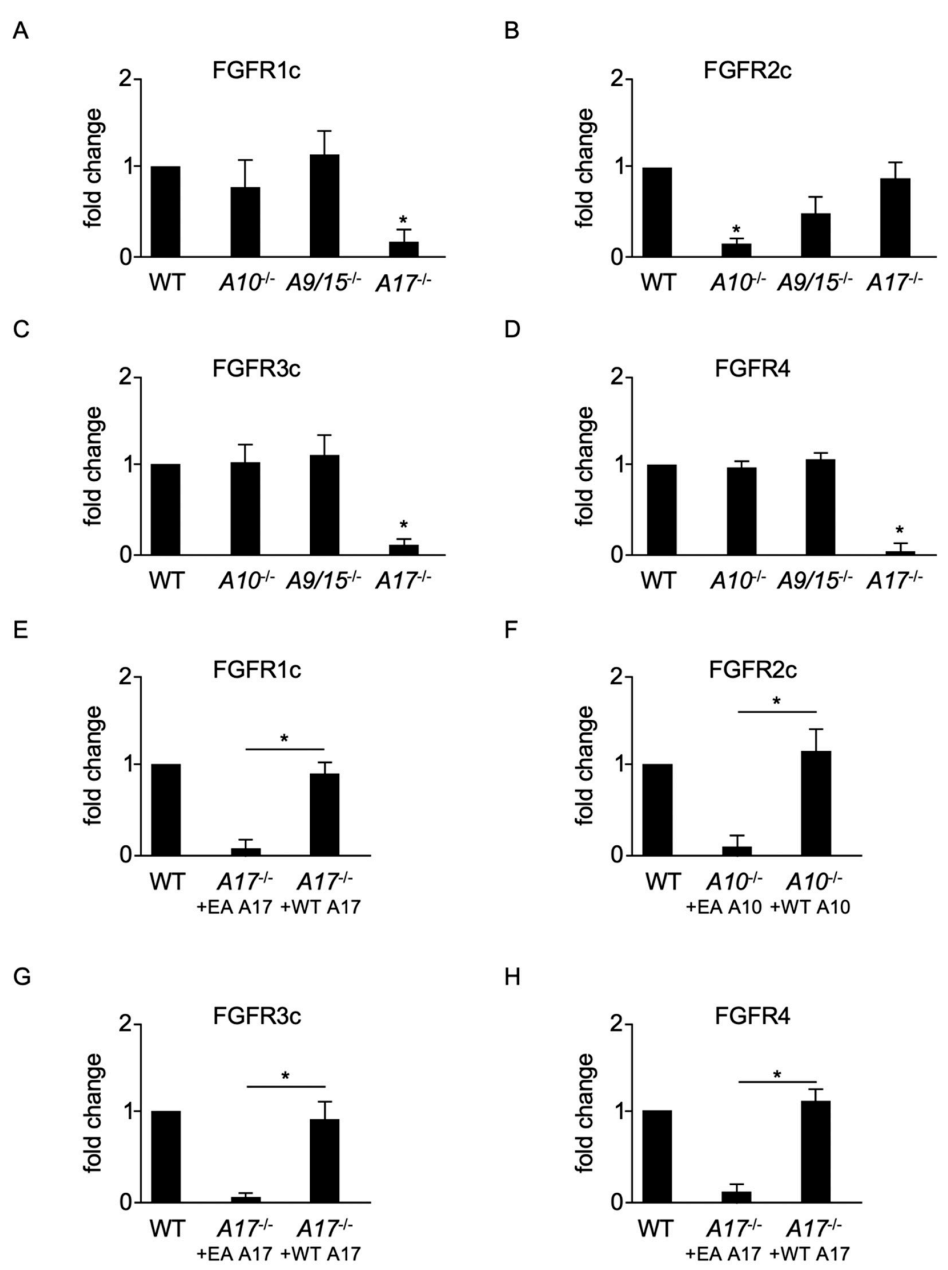
Constitutive shedding of membrane-anchored FGFRs 1–4 is mediated by ADAMs 10 and 17. Shedding of AP-tagged FGFRs 1c (**A**), 3c (**C**), and 4 (**D**) from wild type (WT), *ADAM10*^−/−^, *ADAM9/15*^−/−^, or *ADAM17*^−/−^ mEFs was similar in WT, *ADAM10*^−/−^, and *ADAM9/15*^−/−^ cells, whereas constitutive shedding of FGFRs 1c, 3c, and 4 was strongly reduced in *ADAM17*^−/−^ cells. In contrast, shedding of FGFR2c (**B**) was similar in WT, *ADAM9/15*^−/−^, and *ADAM17*^−/−^ cells but reduced in *ADAM10*^−/−^ cells. *ADAM17*^−/−^ (**E**,**G**,**H**) or *ADAM10*^−/−^ (**F**) mEFs were co-transfected with FGFR1c (**E**), 2c (**F**), 3c (**G**), and 4 (**H**) alone or together with ADAM10 harboring an inactivating point mutation in its catalytic site (*A10*^−/−^ + EA A10) or WT ADAM10 (*A10*^−/−^ + WT A10), and the shedding of FGFRs was compared to that observed in FGFR-transfected WT mEFs. The decreased shedding of FGFRs 1c, 3c, and 4 in *ADAM17*^−/−^ cells transfected with the inactive ADAM17 (EA A17) compared to WT mEFs could be rescued by co-transfection with WT ADAM17 (WT A17, **E**,**G**,**H**). Similarly, the decreased shedding of FGFR2 could be rescued by co-transfection with WT A10 (**F**). (*) indicates a significant reduction in constitutive shedding activity (**A**–**D**), or that the release of FGFR was significantly increased in cells co-transfected with WT compared to inactive EA-expressing cells (**E**–**H**). *n* = 3, ±SEM; analysis via one-way ANOVA; * *p* ≤ 0.05.

To further characterize the role of different ADAMs, we examined FGFR shedding in a genetically defined system. We compared FGFR release in a panel of different FGFR-transfected mouse embryonic fibroblasts (mEFs) deficient for either ADAM10, ADAMs 9/15, or ADAM17 with WT cells. Constitutive shedding of FGFRs 1c (Figure 3A), 3c (Figure 3C), and 4 (Figure 3D) was unaffected in *ADAM10*^−/−^ and *ADAMs 9/15*^−/−^ mEFs and comparable with WT mEFs. In contrast, *ADAM17*^−/−^ cells showed significantly reduced generation of soluble N-terminal fragments (Figure 3A,C,D). Moreover, constitutive shedding of FGFR2c was diminished in *ADAM10^−/−^* cells (Figure 3B), but not significantly affected in *ADAMs 9/15*^−/−^ or *ADAM17*^−/−^ mEFs (Figure 3B). To exclude heterogeneity in cell lines [32], we confirmed the essential roles of ADAMs 10 and 17 for constitutive FGFR shedding in two different independently derived ADAM-deficient cell lines. Finally, we confirmed by “gain-of-function” experiments that the defect in FGFR1c (Figure 3E), 3c (Figure 3G), and 4 (Figure 3H) shedding in *ADAM17*^−/−^ cells was indeed due to lack of ADAM17. Transient transfection of WT ADAM17 (WT A17) rescued constitutive shedding in *ADAM17*^−/−^ mEFs. Similarly, we found that the defect in FGFR2c shedding in *ADAM10*^−/−^ cells was due to lack of ADAM10 (Figure 3F).

### 2.3. Shedding of Membrane-Anchored FGFRs Can Be Induced by the Protein Kinase C Activator PMA and the Calcium Ionophore Ionomycin

In general, shedding of proteins can occur in a constitutive and regulated fashion. ADAM17 has been implied in the regulated shedding of many membrane-bound proteins [17,33], and we therefore set out to analyze in more detail regulated shedding of FGFRs.

**Figure 4:**
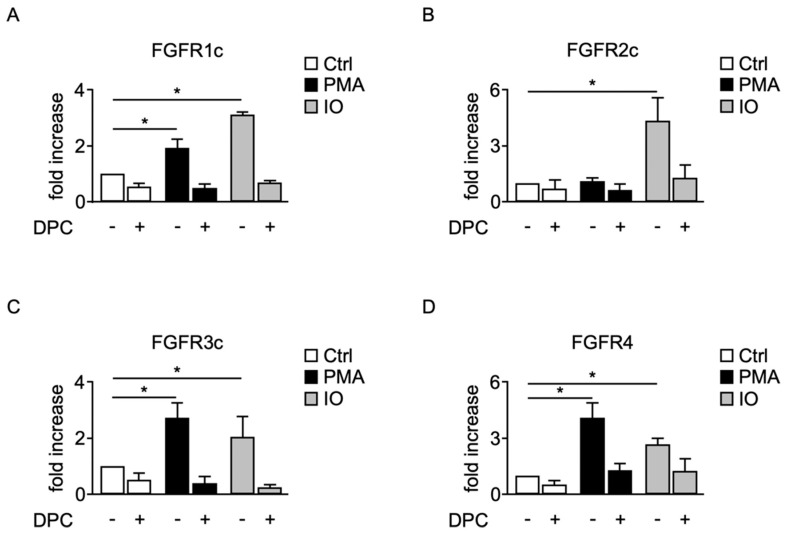
Release of soluble FGFR is induced by the protein kinase C activator PMA and the calcium ionophore ionomycin. Shedding of FGFRs 1c (**A**), 2c (**B**), 3c (**C**), and 4 (**D**) from COS-7 cells incubated with phorbol 12-myristate 13-acetate (PMA, 25 ng/mL), ionomycin (IO, 2.5 μM), with or without the metalloprotease inhibitor DPC333 (DPC, 5μM). Shedding of FGFRs 1c, 3c, and 4 could be stimulated by PMA and IO, whereas shedding of FGFR2c was stimulated by IO but not by PMA. (*) indicates release of FGFR was significantly increased in cells treated with IO or PMA compared to untreated cells. *n* = 3, ±SEM; analysis via two-way ANOVA; * *p* ≤ 0.05.

Stimulation of protein kinase C (PKC) using phorbol ester phorbol-12 myristate 13-acetate (PMA) as well as ionomycin (IO), an agent that promotes shedding through stimulation of calcium influx [34], strongly induced shedding of FGFR1c in COS-7 cells and could be blocked by DPC (Figure 4A). Similar results were obtained with FGFR3c or FGFR4 transfected into COS-7 cells (Figure 4C,D). Identical experiments with FGFR2c showed constitutive shedding with no significant stimulation by PMA, but strong stimulation by IO via DPC-sensitive activity (Figure 4B). These data indicate that cleavage of FGFRs is regulated by several signaling pathways, including PKC and calcium-activated pathways.

### 2.4. Induced Shedding of Membrane-Anchored FGFRs Depends on ADAMs 10 and 17

**Figure 5:**
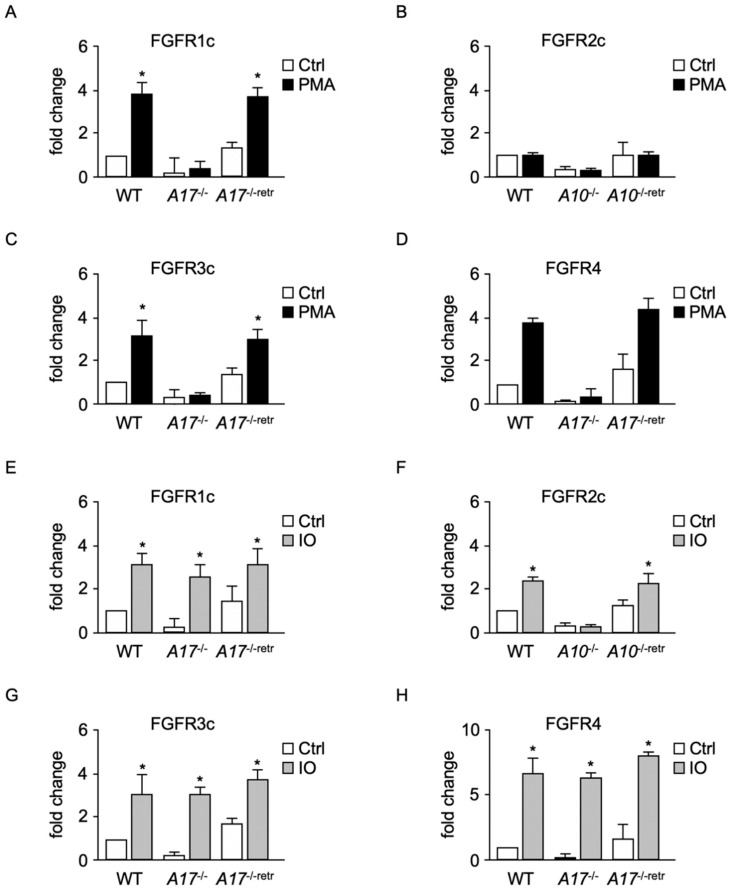
Proteolytic cleavage of membrane-anchored FGFRs in response to activators of metalloproteases depends on ADAMs 10 and 17. *ADAM17*^−/−^ (**A**,**C**–**E**,**G**,**H**) or *ADAM10*^−/−^ (**B**,**F**) fibroblasts were co-transfected with FGFRs 1c (**A**,**E**), 2c (**B,F**), 3c (**C**,**G**), and 4 (**D**,**H**) alone, together with the inactive EA mutant of ADAM17 (*A17*^−/−^, **A**,**C**–**E**,**G**,**H**), inactive EA ADAM10 mutant (*A10*^−/−^, **B**,**F**), wild type (WT) ADAM17 (*A17*^−/−retr^, **A**,**C**–**E**,**G**,**H)**, or WT ADAM10 (*A10*^−/−retr^, **B**,**F**) and treated with or without phorbol 12-myristate 13-acetate (PMA, 25 ng/mL) (**A**–**D**) or ionomycin (IO, 2.5 μM) (**E**–**H**). (*) indicates release of FGFR was significantly increased in cells treated with PMA or IO compared to untreated cells. *n* = 3, ±SEM; analysis via two-way ANOVA; * *p* ≤ 0.05.

PMA stimulation of FGFRs 1c, 3c, and 4c shedding indicated a possible role for ADAM17 [34], whereas stimulation of FGFR2c-sheddase by IO, but not PMA, matched the properties of an ADAM10 substrate [34,35]. Shedding of FGFRs 1c, 3c, and 4 from WT mEFs was stimulated by PMA, whereas less constitutive and almost no PMA-stimulated shedding of FGFRs 1c, 3c, and 4 was seen in *ADAM17^−/−^* mEFs (Figure 5A,C,D). PMA-stimulated shedding of FGFRs 1c, 3c, and 4 from *ADAM17^−/−^* mEFs could be rescued by ADAM17, but not by its inactive form. In contrast, PMA did not show any significant effects on shedding of FGFR2c from WT or *ADAM10^−/−^* mEFs (Figure 5B). Similar experiments showed IO-stimulated shedding of all four FGFRs from WT mEFs (Figure 5E–H), but only little IO-stimulated shedding of FGFR2c from *ADAM10^−/−^* mEFs (Figure 5F). Shedding of FGFR2c from *ADAM10^−/−^* mEFs was enhanced by ADAM10, but not by its inactive form (Figure 5F).

### 2.5. Soluble Forms of FGFR2 Inhibit FGF7-Induced Epithelial Cell Migration

**Figure 6:**
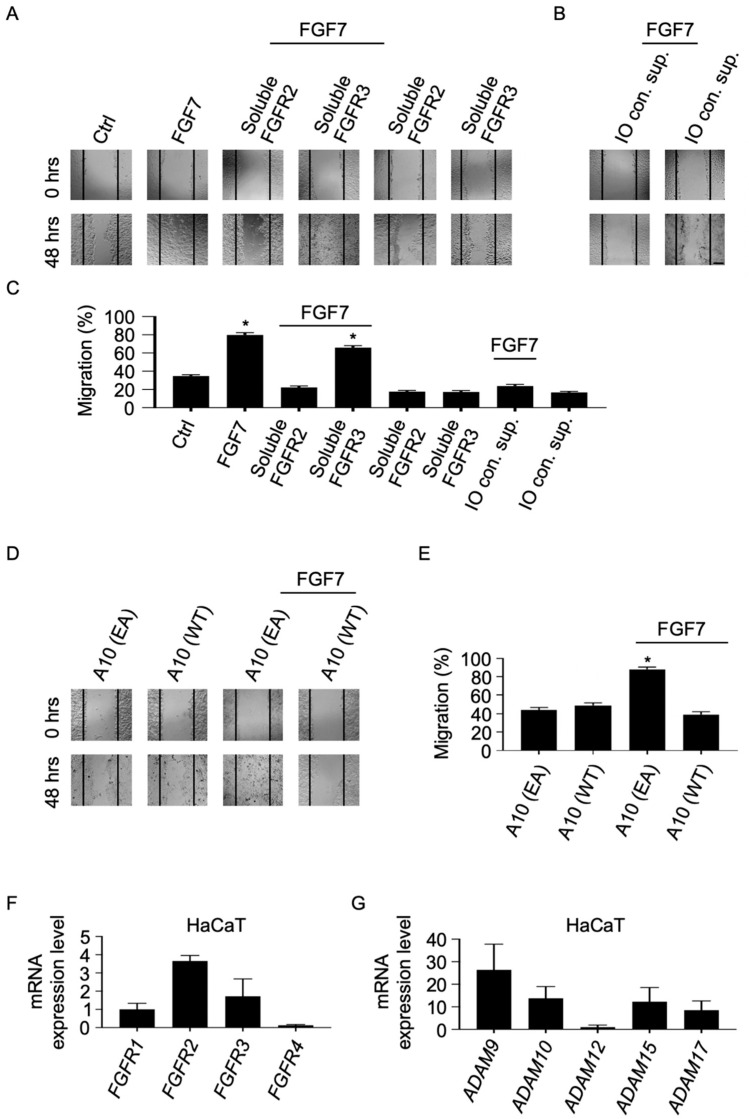
Soluble FGFR2 reduces FGF7-stimulated epithelial cell migration. HaCaT cells were treated with or without FGF7 (50 ng/mL) in the presence or absence of recombinant soluble FGFR2 (50 ng/mL) or FGFR3 (50 ng/mL) (**A**). A 10 cm plate with HaCaT cells was treated with 1 μM IO for 1 h, and a volume of 1 mL of these conditioned supernatants was removed and incubated for 24 h with HaCaT cells in the presence or absence of FGF7 (50 ng/mL) (**B**). HaCaT cells were transfected with wild type (WT) ADAM0 (A10) or the inactive mutant (EA) A10 and cultured in the presence or absence of recombinant FGF7 (50 ng/mL) (**D**). A cell-free area was introduced with a pipette tip, and micrographs were taken at 0 and 24 h after scratch wounding (**A**,**B**,**D**). One representative of three independent experiments is shown. Scale bar, 100 μm. Quantification of the results of three separate in vitro scratch wound healing assays is shown in (**C**,**E**). Quantification of the mRNA levels of FGFRs 1–4 (**F**) and ADAMs 9, 10, 12, 15, and 17 (**G**) in HaCaT cells by real-time qRT-PCR. (*) indicates a significant increase in cell migration compared with ctrl (**C**) or A10 EA (**E**) samples. *n* = 3, ±SEM; analysis via one-way ANOVA; * *p* ≤ 0.05.

Previous studies have shown that soluble forms of FGFR family members can act as endogenous signaling inhibitors by either acting as traps of their respective ligands or by blocking the membrane-bound receptor’s ligand-binding domain [10,36,37,38]. To assess the functional relevance of metalloprotease-mediated shedding of FGFRs, we evaluated the effect of soluble FGFR2 in an in vitro scratch wound healing assay with HaCaT cells. In this epithelial cell line, FGF7-induced migration depends on activation of FGFR2 [16]. Untreated HaCaT cells did not repair scratch wounds after 48 h, but treatment with FGF7 led to a nearly complete closure of the wound in 48 h (Figure 6A). FGF7-stimulated migration of HaCaT cells could be blocked by addition of recombinant soluble FGFR2 but not by soluble FGFR3, which does not bind to FGF7 [39,40]. To determine whether endogenous soluble FGFR2 could affect FGF7-stimulated epithelial cell migration, we induced shedding of FGFR2 in HaCaT cells with IO, and then collected and added these conditioned supernatants to HaCaT cells in the presence or absence of FGF7. Cell migration of FGF7-treated HaCaT cells could be inhibited by addition of IO-conditioned supernatants while IO-conditioned supernatants alone did not block basal migration of HaCaT cells (Figure 6B; a quantification of the results from three separate experiments is shown in Figure 6C). When similar experiments were performed in HaCaT cells overexpressing wild type (WT) ADAM10 (A10), FGF7-stimulated cell migration was reduced, suggesting an ADAM10-mediated decrease in FGFR2 cell surface presentation in these cells. However, overexpression of a catalytically inactive ADAM10 mutant (A10 EA) did not affect FGF7-induced migration in these cells (Figure 6D; a quantification of the results from three separate experiments is shown in Figure 6E). Finally, we established the expression patterns of catalytically active ADAMs and FGFRs in HaCaT cells by real-time quantitative reverse transcription PCR (real-time qRT-PCR). We found that all four *FGFRs* were expressed in these cells. We detected substantial levels of *FGFR2* and *FGFR3* and similarly of *FGFR1*, albeit at lower levels. By contrast, we only detected very low levels of *FGFR4* expression (Figure 6F). In addition, we detected similar expression levels of ADAMs 9, 10, 15, 17 as well as lower levels of ADAM12 in these cells (Figure 6G).

## 3. Discussion

FGFRs are a family of receptor tyrosine kinases expressed on the cell surface on many different cell types and regulate key cell behaviors, such as vascular and skeletal development during embryogenesis, as well as regulation of angiogenesis and wound healing in adults [41,42]. In addition to their membrane-anchored forms, secreted, soluble forms of FGFRs have long been documented in multiple biological fluids and presumably regulate the biological activities of the FGF family of proteins in vivo, both in circulation and the extracellular matrix [12,13,14,15]. Previous studies have shown that soluble forms of members of the FGFR family can be produced by translation of alternatively spliced transcripts as well as through posttranslational ectodomain shedding of membrane-anchored receptors [15], but little is known about the proteolytic system responsible. This prompted us to identify and characterize novel proteases that can participate in ectodomain shedding of this subfamily of receptor tyrosine kinases.

Our present study demonstrates that two distinct metalloproteases, ADAM10 and ADAM17, are critically involved in controlling the release of soluble FGFRs 1c, 2c, 3c, and 4 by membrane proximal cleavage. Initial evidence that an ADAM metalloprotease is a candidate for constitutive cleavage of FGFRs was obtained in inhibition studies with the hydroxamate-based inhibitor DPC that has been shown to inhibit ADAM17 as well as ADAM10 [34,43,44]. In accordance with other reports implicating the role of ADAMs in shedding of membrane bound proteins from many cell surfaces [1,45,46,47], DPC333 reduced constitutive release of FGFRs from WT fibroblasts and COS-7 cells. We confirmed these findings by demonstrating that constitutive shedding of FGFRs 1c, 3c, and 4 was nearly completely abolished in *ADAM17*^−/−^ fibroblasts, while the constitutive cleavage of FGFR2c depended on ADAM10. Thus, our data represent the first evidence in a genetically defined system that ADAMs 10 and 17 are responsible for the constitutive as well as induced proteolysis of FGFRs 1–4. Because ADAMs 10 and 17 are ubiquitously expressed [48,49], it is tempting to speculate that they may participate in the functional regulation of these four FGFRs in development and in diseases such as achondroplasia and cancer [50,51,52].

ADAM10- or ADAM17-dependent shedding can be posttranslationally upregulated by phorbol esters (ADAM17) or the calcium ionophore IO (ADAMs 10 and 17) within 30 min [25,34]. Our results identified ADAM10 as the relevant IO-stimulated sheddase for FGFR2c in cell-based assays. Furthermore, our results indicate that ADAM10 does not make a major contribution to PMA-stimulated release of FGFRs 1c, 3c, and 4 in the cells tested here. This is consistent with the notion that different stimuli activate different ADAMs [17,35]. In light of our loss-of-function studies, which demonstrated that ADAM17 is required for the constitutive and PMA-stimulated shedding of FGFRs 1c, 3c, and 4, it was unexpected to find activity in *ADAM17*^−/−^ cells that was able to efficiently process these three FGFRs upon stimulation with IO. Interestingly, a previous study has identified ADAM10 as a sheddase that can, in principle, release ADAM17 substrates such as TGFα and TNF almost as efficiently as their primary sheddase but only if ADAM17 is inactive [53]. Our findings suggest that FGFRs 1c, 3c, and 4 are also shed by another metalloprotease, presumably ADAM10, from *ADAM17*^−/−^ cells when stimulated with IO. Additional studies in *ADAM10/17* double-deficient cells will be necessary to further explore the requirement for ADAM10 for the IO-stimulated shedding of FGFRs 1c, 3c, and 4.

While our study focused on the proteolytic processing of the FGFR c splice isoforms, in future studies, it will be important to determine if the b splice isoforms of these receptors are shed by the same proteases in a similar manner. In common with our current findings, we have previously shown that the b splice isoform of the FGFR2 is also shed by ADAM10 [16], suggesting that the C-terminal half of the third Ig-like domain of the receptor might not affect the substrate recognition, at least in the case of FGFR2.

Previous studies have shown that FGFRs can be shed from the plasma membrane and are found in a secreted, soluble form [15], yet little is known about how soluble FGFRs could regulate biological activity of FGFs. Our results, that FGF7/FGFR2-dependent cell migration in keratinocytes can be inhibited by soluble forms of FGFR2, suggest that these forms may control the bioavailability of FGFs. However, some limitations should be noted. Although the stimulation of keratinocytes with IO presumably leads to an increased shedding of FGFR2 into conditioned medium, it is possible that other molecules in these conditioned supernatants could also potentially affect FGF7/FGFR2-mediated signaling and migration of these cells. Moreover, it is possible that the remaining amount of IO in the conditioned supernatants increases shedding of membrane bound FGFR2 during the time of migration assay and therefore further reduces FGF7-dependent activation of FGFR2. However, the addition of recombinant FGFR2 suggests that soluble forms of FGFR2 might be sufficient to block FGFR2 function in vitro, so it is tempting to speculate that ADAM-mediated shedding of FGFRs can have a crucial role in regulating FGFR function at a posttranslational level in vivo.

While the physiological relevance of ADAMs 10 and 17 in processing FGFRs remains to be determined, it is tempting to speculate that these metalloproteases could contribute to the downregulation of the membrane-anchored form of the receptor, at least in cells or tissues in which both proteins are highly expressed. Additional studies in conditional *ADAM10*- and *ADAM17*-deficient mice will further aid in dissecting the role of these metalloproteases in regulating FGFR-mediated cell growth and division in vivo.

## 4. Materials and Methods

### 4.1. Cell Lines and Reagents

Simian virus large-T-antigen-immortalized mouse embryonic fibroblasts (mEFs) were generated from wild type (WT) or *ADAM9/15*^−/−^ E13.5 embryos and cultured as described previously [17,54]. In addition to *ADAM9/15*^−/−^ mEFs, we also used *ADAM17*^−/−^ mEFs [34,55] as well as WT controls of mixed genetic background (129Sv/C57Bl6) to generate corresponding immortalized mEFs [17]. *ADAM10*^−/−^ fibroblast cell lines derived from E9.5 embryos have been described previously [32]. COS-7 cells were from the American Type Culture Collection and experiments were partially conducted in the laboratory of Dr. Carl P. Blobel of the Hospital for Special Surgery, New York, NY, USA. All cells were grown in Dulbecco’s modified Eagle medium supplemented with 1% penicillin/streptomycin and 5% fetal calf serum. All reagents were from MilliporeSigma (Burlington, MA, USA) unless otherwise indicated. Ionomycin (IO) and phorbol 12-myristate 13-acetate (PMA) were obtained from R&D Systems; Minneapolis, MN, USA. The metalloprotease inhibitor DPC333 was kindly provided by Dr. Carl P. Blobel [43,44].

### 4.2. Expression Vectors

The expression vectors for ADAMs 9, 10, 12, 15, and 17 have been validated for cell surface localization and proteolytic activity previously [21,25,35,56,57]. In addition, comparable gene expression levels in transiently transfected COS-7 cells with these vectors (Figure 2E–H) were confirmed by mRNA real-time qRT-PCR (Figure S1). cDNAs encoding alkaline phosphatase (AP)-tagged FGFRs 1–4 were constructed by re-cloning full-length human FGFR 1–4 cDNAs into the *Xho*I and/or *Xba*I sites of pKitl2APtag5 vector [23] in frame, as described previously [58]. In both cases, an AP-tag was attached to the N-terminus of the full-length WT protein. All constructs were sequenced to confirm that desired mutations or deletions had been obtained, and to rule out other mutations.

### 4.3. Transfection and Ectodomain Shedding Assays

Mouse embryonic fibroblasts or COS-7 cells were transiently transfected with Lipofectamine 2000 (Thermo Fisher Scientific, Waltham, MA, USA), per manufacturer’s protocol, using ADAM WT or catalytically inactive (EA) plasmids at a concentration of 1.5 μg per well of a 12-well plate, unless otherwise indicated, and FGFRs 1 to 4-AP vectors at 1 μg. Shedding assays were performed the day after transfection [17,35,53,59]. For shedding assays including inhibitors, cells were preincubated with or without inhibitors for 5 to 15 min. For stimulation experiments, cells were washed with Opti-MEM medium (Thermo Fisher Scientific, Waltham, MA, USA) for 1 h followed by incubations with indicated stimulus for 45 min. Constitutive shedding was measured after 2 h of incubation. Evaluation of AP activity in supernatants and cell lysates by colorimetric assays was performed as described previously [17,59]. Briefly, the relative shedding activity of a given ADAM protease toward a given FGFR substrate was calculated to normalize for variability in transfection efficiency and for differences in cDNA expression between samples by determining the ratio of AP-tagged FGFR shed into the supernatant to the remaining FGFR precursor in the cell lysate. No AP activity was present in conditioned media of non-transfected cells.

### 4.4. In-Gel Alkaline Phosphatase Assay

In-gel detection of alkaline phosphatase-labeled membrane proteins was performed as previously described [59,60,61]. Briefly, lysates of cells expressing AP-tagged human AP-tagged FGFRs 1–4 were separated by SDS-PAGE, and AP was renatured by incubating the gel twice for 30 min in 2.5% Triton X-100 (MilliporeSigma, Burlington, MA, USA) and visualized by adding AP substrates nitro blue tetrazolium and 5-bromo-4-chloro-3-indoly phosphate.

### 4.5. In Vitro Scratch Wound-Healing Assays

Cells used for in vitro scratch wound-healing assays were plated in 12-well plates and grown until they reached confluence. A thin scratch wound was introduced by scratching with a 200 μL pipette tip. After washing with phosphate-buffered saline, cells were incubated with or without FGF7, soluble forms of FGFRs 2 and 3, or IO as indicated. Images were taken using a digital transmitted light, inverted imaging system (EVOS XL core, Invitrogen, Carlsbad, CA, USA), and NIH Image J software (https://imagej.nih.gov/, accessed on 17 December 2020) was used for quantification of scratch wound assays [62].

### 4.6. Real-Time qRT-PCR

Total RNA from HaCaT cells was extracted using the RNeasy Plus Mini kit (Qiagen, Germantown, MD, USA), and cDNA was produced with 500 ng RNA using a reverse transcription kit (ProtoScript^®^ First Strand cDNA Synthesis Kit, New England Biolabs, Rowley, MA, USA) following manufacturer’s protocol. RNA quantity and quality were measured with a NanoDrop ND-1000 spectrophotometer (Thermo Fisher Scientific, Waltham, MA, USA). Pre-verified qPCR primers for *FGFR1–4* and *ADAMs 9*, *10*, *12*, *15*, and *17* were purchased from MilliporeSigma (Burlington, MA, USA) (https://www.kicqstart-primers-sigmaaldrich.com, accessed on 17 December 2020). Gene transcripts were measured in triplicates on a real-time PCR detection system (Bio-Rad, Des Plaines, IL, USA) using a PerfeCTa SYBR^®^ Green FastMix (Quantabio, Beverly MA, USA) and normalized to the reference gene *Beta-actin*. Thermocycling conditions were set as follows: 1 cycle (95 °C for 5 min), 40 cycles (95 °C for 15 s, 58 °C for 60 s).

### 4.7. Statistical Analysis

Data analysis was performed using GraphPad Prism 8.0 (GraphPad Software, San Diego, CA, USA). All values are expressed as means ± standard error of the mean (SEM). One or two-way analysis of variance (ANOVA) testing was performed. Multiple parametric statistical comparisons between experimental groups versus a control group were accomplished by Dunnett’s method. All pairwise multiple comparison procedures were performed via Bonferroni’s multiple comparison test. Statistics following a Student’s *t* distribution were generated using a *t*-test. Standard error values indicate variation between mean values obtained from at least three independent experiments. *P*-values ≤ 0.05 were considered as statistically significant.

## 5. Conclusions

In summary, this study demonstrates that membrane-anchored members of the FGFR family are shed from various cell types, including COS-7 cells and mEFs, and identifies the responsible sheddases. The release of soluble forms of FGFR should decrease their availability on cells and simultaneously generate soluble decoys that could regulate the biological activity of members of the FGF family of growth factors, similar to other soluble RTKs.

## Data Availability

Data is contained within the article and supplementary material.

## Supplementary Materials

The following is available online at https://www.mdpi.com/1422-0067/22/6/3165/s1.

## Abbreviations

ADAM9	A disintegrin and metalloprotease 9
ADAM10	A disintegrin and metalloprotease 10
ADAM15	A disintegrin and metalloprotease 15
ADAM17	A disintegrin and metalloprotease 17
AP	Alkaline phosphatase
DPC333	DPC333 ((2R)-2-((3R)-3amino-3(4-[2-methyl-4-quinolinyl)methoxyl] phenyl)-2-oxopyrrolidinyl)-N-hydroxy-4-methylpentanamide))
EGFR	Epidermal growth factor receptor
FGFR1	Fibroblast growth factor receptor 1
FGFR2	Fibroblast growth factor receptor 2
FGFR3	Fibroblast growth factor receptor 3
FGFR4	Fibroblast growth factor receptor 4
FL	Full-length
IO	Ionomycin
mEF	Mouse embryonic fibroblast
NTF	N-terminal fragment
PMA	Phorbol 12-myristate 13-acetate
RTK	Receptor tyrosine kinase
WT	Wild type
