# Supplementary material for: Members of the Fibroblast Growth Factor Receptor Superfamily Are Proteolytically Cleaved by Two Differently Activated Metalloproteases
Source: Int J Mol Sci. 2021 Mar 20;22(6):3165. doi: 10.3390/ijms22063165 (PMC8003738; doi:10.3390/ijms22063165)
Supplement: Supplementary file 1 [file ijms-22-03165-s001.pdf]

Figure S1

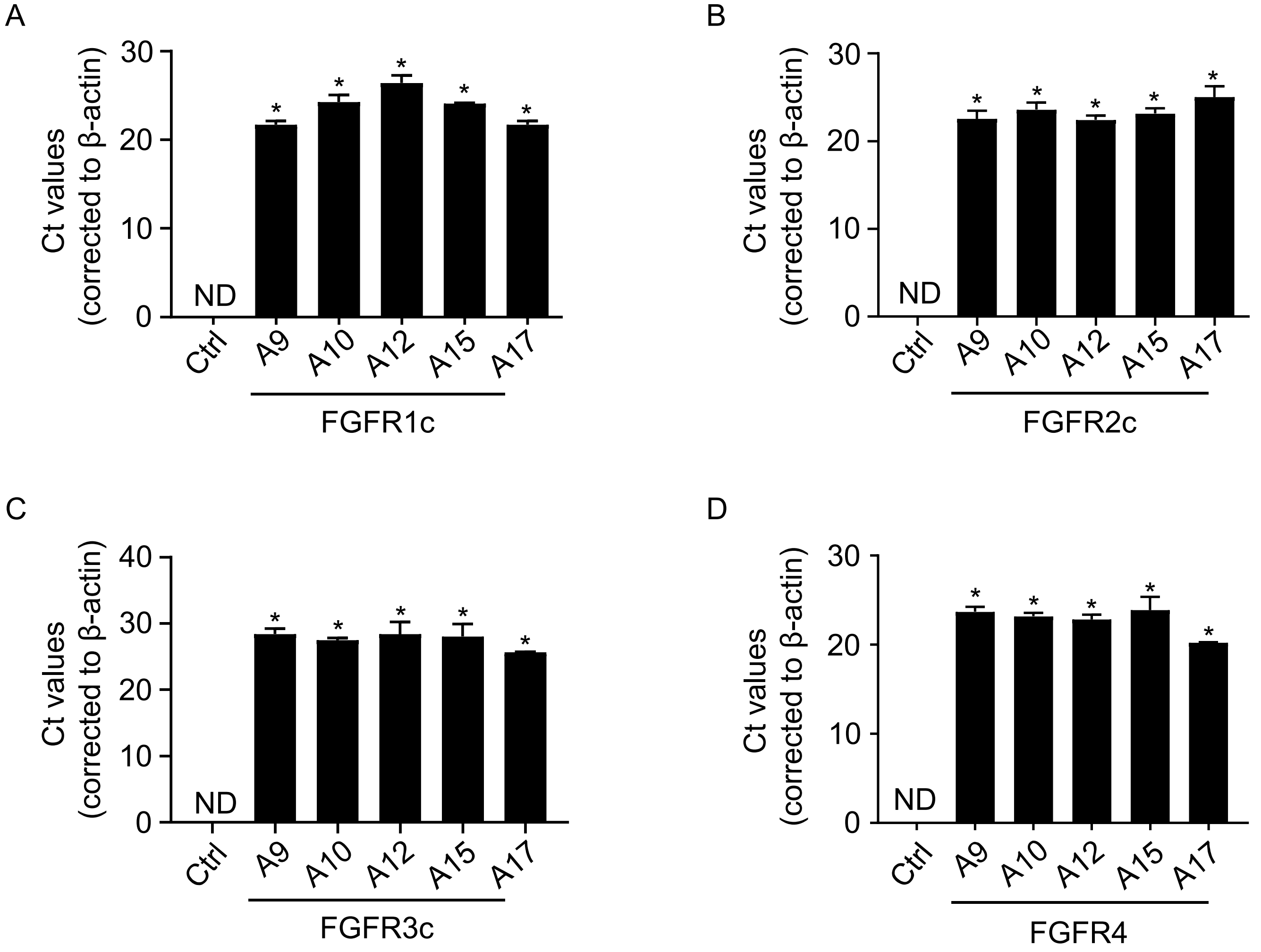

Figure S1. Gene expression levels of five transiently overexpressed ADAMs in COS-7 cells. Overexpression of ADAMs 9 (A9), 10 (A10), 12 (A12), 15 (A15), or 17 (A17) with FGFRs 1c (**S1A**), 2c (**S1B**), 3c (**S1C**) and 4 (**S1D)** in COS-7 cells. (*) indicates significant increase in ADAMs 9, 10, 12, 15, or 17 mRNA expression in transfected COS-7 sample compared with untransfected sample. $n = 3$; values are ± s.e.m; Student's t-test; $*P \leq 0.05$.
